# The effect of Teach-back method education on the control of asthma and family care pressure of patients in Iran*

**DOI:** 10.17533/udea.iee.v40n1e04

**Published:** 2022-03-29

**Authors:** Mohammad Imanipour, Zahra Molazem, Mahnaz Rakhshan, Mohammad Javad Fallahi, Amir Mohammad Atashin Sadaf

**Affiliations:** Student Research Committee. Email: mohammad.imani2014@gmail.com. mohammad.imani2014@gmail.com; 2 Professor of Nursing. Email: molazem@sums.ac.ir. Corresponding author. molazem@sums.ac.ir; 3 Associate Professor of Nursing. Email: rakhshanm@sums.ac.ir rakhshanm@sums.ac.ir; 4 Assistant Professor of Medicine. Email: fallahimj@sums.ac.ir fallahimj@sums.ac.ir; 5 M.Sc Student in Medical Surgical Nursing. Zanjan University of Medical Sciences, Zanjan, Iran. Email: atashinsadaf.1995@gmail.com Zanjan University of Medical Sciences Zanjan University of Medical Sciences Zanjan Iran atashinsadaf.1995@gmail.com; 6 Shiraz University of Medical Sciences, Shiraz, Iran. Shiraz University of Medical Sciences Shiraz Iran

**Keywords:** asthma, teach-back communication, caregiver burden., asma, método teach-back, carga del cuidado., asma, comunicação para apreensão de informação, fardo do cuidador.

## Abstract

**Objective.:**

To investigate the effect of teach-back education on patient asthma control and family care pressure of patients with asthma.

**Methods.:**

The present study is a clinical trial and the study population was patients referred to Shahid Faghihi and Shahid Motahhari clinics in Shiraz, Iran. 58 patients with asthma and their caregivers were randomly assigned to the intervention and control groups, for a total of 29 subjects in each group. In the intervention group: the teach-back method was delivered individually to the patient and his or her primary caregiver in three sessions of approximately 60 minutes at one-day intervals. each session included presentations, practical techniques and a booklet. In this study, patients and caregivers in the control group were not trained. Before the intervention, 4 and 8 weeks after the intervention, asthma control test and spirometry test were performed to evaluate asthma control; Also, before the intervention and 8 weeks after the intervention, Zarit test was performed to evaluate the care burden.

**Results.:**

The findings of repeated measures tests showed that, compared to the control group, the intervention group obtained a greater increase in the vital capacity index (*p*=0.028) and in the disease control score (*p*=0.001), as well as a reduction in the burden of care on family members (*p*<0.001).

**Conclusion.:**

The present study showed that teaching asthma related topics to the patient and her caregiver along with the follow-up and supervision of the nurse improves the asthma control of the patient and also reduces the caregiver pressure.

## Resumen

**Objetivo.:**

Investigar el efecto de la educación con el método teach-back sobre el control del asma y la carga del cuidado familiar de estos pacientes.

**Métodos.:**

Ensayo clínico cuya población de estudio fueron los pacientes remitidos a las clínicas Shahid Faghihi y Shahid Motahhari en Shiraz, Irán. Se asignaron aleatoriamente 58 pacientes con asma y sus cuidadores a los grupos de intervención y control, con un total de 29 díadas en cada grupo. Las personas del grupo de intervención recibieron formación con el método teach-back que se impartió individualmente al paciente y a su cuidador principal en tres sesiones de aproximadamente 60 minutos en tres días consecutivos. Cada sesión incluía presentaciones, técnicas prácticas y un folleto. Los pacientes y cuidadores del grupo de control no recibieron formación. Antes de la intervención y a las 4 y 8 semanas después de la misma, se aplicaron las escalas de control del asma y la de Zarit para evaluación de la carga del cuidado y, además se practicó una espirometría.

**Resultados.:**

Las pruebas de medidas repetidas entre los grupos de estudio mostraron que, comparando con el grupo control, el grupo de intervención obtuvo mayor aumento del índice de capacidad vital (*p*=0.028) y del puntaje de control de la enfermedad (*p*=0.001), además, se redujo la carga del cuidado en los familiares (*p*<0.001).

**Conclusión.:**

El presente estudio mostró que con la enseñanza de temas relacionados con el asma al paciente y a su cuidador, junto con el seguimiento y la supervisión de la enfermera, se mejora el control del asma en el paciente y también se reduce la carga del cuidador.

## Resumo

**Objetivo.:**

Investigar o efeito da educação com o método teach-back sobre o controle da asma e a carga do cuidado familiar destes pacientes.

**Métodos.:**

Ensaio clínico no qual a população de estudo foram os pacientes enviados às clínicas Shahid Faghihi e Shahid Motahhari em Shiraz, Irã. 58 pacientes com asma e seus cuidadores foram designados aleatoriamente aos grupos de intervenção e controle, com um total de 29 díade em cada grupo. As pessoas do grupo da intervenção receberam formação com o método teach-back que se transmitiu individualmente ao paciente e ao seu cuidador principal em três sessões de aproximadamente 60 minutos em três dias consecutivos. Cada sessão incluía apresentações, técnicas práticas e um folheto. Os pacientes e cuidadores do grupo de controle não receberam formação. Antes da intervenção e às 4 e 8 semanas depois dela, se aplicaram as escalas de controle da asma e a de Zarit para avaliação da carga do cuidado e, além disso se praticou uma espirometria.

**Resultados.:**

As provas de medidas repetidas entre os grupos de estudo mostraram que, comparando com o grupo de controle, o grupo de intervenção obteve maior aumento do índice de capacidade vital (*p*=0.028) e da pontuação de controle da doença (*p*=0.001), ademais de que se reduziu a carga do cuidado nos familiares (*p*<0.001).

**Conclusão.:**

O presente estudo mostrou que o ensino de temas relacionados com a asma ao paciente e ao seu cuidador, junto com o seguimento e a supervisão da enfermeira, se melhora o controle da asma no paciente e também se reduz a carga do cuidador.

## Introduction

Today, chronic diseases are recognized as the most important health problem, especially in developing countries. Asthma is a common chronic inflammatory disease of the respiratory tract characterized by a variety of symptoms, including airway obstruction and bronchitis. Asthma is currently considered as one of the most serious health problems.(1,2) In a 2014 report on the global prevalence of asthma states that there are approximately 300 million people with asthma worldwide and that it is expected to increase by about 33% to 20 million until the year 2025.(3) Also, the prevalence of asthma and chronic bronchitis in Iran has been reported from 4.8 to 5.6%.(4)

Quality of life is considered as an important criterion for studying chronic diseases. This means feeling good in physical, mental and social aspects. Accordingly, quality of life is used to assess community health needs, assess the social impact of the disease, identify at-risk individuals, implement appropriate health policies, and allocate health resources. Because asthma is a chronic disease with a high cost of treatment; it is difficult to manage, as a result, it reduces the quality of life of patients and their families.(5,6) Despite many recommendations about the role of disease prevention and control through education of correct behaviors, many studies still acknowledge that the disease is slow and difficult to control. This disease is not curable, but with a series of treatment and care measures, it can be controlled to some extent.(7,8)

Educating patients increases their understanding of the disease, treatment, and can have positive effects on the patient's performance, physical condition, quality of life, adaptation, and reduction of emotional problems.(9) One of the most effective methods to improve the understanding of education is the teach-back method. This method is a comprehensive, multidisciplinary, evidence-based strategy used to understand and retain information. Health care organizations have endorsed this approach as an effective way to ensure that health care information is understood and reduce the risk of patients misunderstanding essential information in clinical situations.(10,11) In the teach-back method, essential information is explained to the patient in a way that the patient understands and is an educational method to ensure patients' understanding. The purpose of the feedback method is to provide effective learning at the patient literacy level. The advantages of using this method are improving the patient's relationship with the treatment staff, increasing patient safety, better evaluation of the educator from the patient, and usability in people with low literacy levels.(12,13) Therefore, the aim of this study was to investigate the effect of teach-back education on patient asthma control and family care pressure of patients with asthma referred to clinics affiliated to Shiraz University of Medical Sciences, Shiraz, Iran. Accordingly, if the intervention performed in this study is effective, its results can be used as an effective way to increase the quality of life and improve asthma control of patients and reduce the stress of caring for their families.

## Methodology

The present study is a clinical trial and the study population was patients referred to Shahid Faghihi and Shahid Motahhari clinics in Shiraz, Iran, who referred to the pulmonary ward in the year 2020. Overall, 58 patients with asthma who had the condition participated in the study.

Patients, inclusion, and exclusion criteria. The number of patiets in each group was 29 patients and 29 primary caregivers. Research units were selected by the available sampling method. Then they were assigned to two groups by random assignment. Additionally, inclusion criteria for patients included: moderate to severe asthma (Based on the doctor's opinion), age range 18 to 60 years, willingness to participate in research and fill out informed consent form, not participating in similar programs during the last 6 months, having the ability to understand based on the researcher's judgment, and the possibility of making telephone calls. On the contrary, exclusion criteria also included; absenteeism in educational classes, impossibility to continue cooperation, having a degree in medical sciences and suffering from other chronic diseases. Inclusion criteria for the patient's primary caregiver include: willingness to participate in research and filling out informed consent form, not participating in similar programs during the last /6 months, having the ability to understand based on the researcher's judgment, not caring for other chronic conditions, possibility Making phone calls and not having a critical or stressful event (such as death of relatives, divorce, illness, or immigration in the last three months). Furthermore, absenteeism in educational classes, the impossibility of continuing cooperation in the study, and having education in the medical sciences group were the exclusion criteria for the main caregiver.

Sample size. Based on the study of York *et al*.,(1) the sample size in this study (*n*=58) also using NCSS software using the mean difference formula between the two groups considering α=0.05, power 80%, mean and standard deviation of group one (0.62 ± 0.05), the mean and standard deviation of group two (0.66 ± 0.05) was determined by considering a drop of 20% in each group:

$N=\frac{{\left(1.96+0.84\right)}^{2}\left(0.05^{2}+0.05^{2}\right)}{{\left(0.66-0.62\right)}^{2}}$

N=Z1-α2+Z1-β2S12+S22X-1-X-22

Data collection tools. Data collection tools included patient demographic information questionnaire, demographic information questionnaire of the main caregiver, asthma control test, lung function test (Spirometry), and ZARIT care pressure questionnaire.

Asthma control test and lung function test (Spirometry). To evaluate the asthma control of patients, asthma control test and lung function test (Spirometry) were used at intervals before the start of the study, 4 and 8 weeks after the intervention. The asthma control test is based on GINA institute criteria and allows patients older than 12 years to assess their asthma control status over 4 weeks.(2) The reliability of asthma control test in various studies has been reported equal to 0.94 and its validity has been confirmed based on the correlations between asthma control test and other tools for measuring asthma recovery status.(3) In the asthma control test, a score of 5 indicates the best position and a score of 1 indicates the worst position of asthma control. The recorded scores were added together to obtain the total score. The total score was used to assess asthma control status includes score 25 (asthma has been completely under control for the past 4 weeks), score 20 to 24 (asthma has been under control for the past 4 weeks), and score 19 or less (has not been controlled in the last 4 weeks).

Lung function test is performed by a specialist technician using ZAN Spirometry device (Germany) in accordance with the principles related to this test. In this study, indicators such as Forced Expiratory Volume in first second (FEV1), Forced Vital Capacity (FVC), FEV1 / FVC ratio and Forced Expiratory Flow rate %25 - %75 (FEF25 -75%) were measured.

Assess the quality of life of patients. To assess the quality of life of patients, the 67-item questionnaire of Marx et al. was used at intervals before and 8 weeks after the intervention. This questionnaire measures quality of life in 5 dimensions: respiratory function, physical activity, mood function, social function and general perception of health. The answer to each question was scored on a 5 degree Likert scale. In this questionnaire, increasing the total score indicates an improvement in the quality of life. Each question has a number of qualitative answers including: ever, rarely, sometimes, most and always. The score of each positive question is between 1 and 5. The option "always" has a score of 5 and the option "never" has a score of 1. In the negative questions, the option "always" has a score of 1 and the option "never" has a score of 5. Responses to each scale were reported to be rated between 0-100 on average. The validity of this questionnaire in terms of content validity has been confirmed by Marx et al. and has a Cronbach's alpha of 94%. Also, Cronbach's alpha of the subscales of this questionnaire has been reported in the range of 0.79 to 0.85.(4) Additionally, the validity of this questionnaire in Iranian society was assessed by Arab et al. (2012); The content validity index was 0.90 and in order to determine the internal reliability, the test-retest method was used and the correlation coefficient was 0.75.(5)

Assess the care burden of family caregivers. To assess the caregiver burden of family caregivers, the 22-item form of Zarit care burden questionnaire was used at intervals before and 8 weeks after the intervention. This questionnaire is about personal, social, emotional and economic pressures. Caregivers' responses for each statement were measured on a Likert scale with five options (never to always) that were scored from 0 to 4, respectively. In response to each question, the study units chose one of the cases never (zero score), rarely (score 1), sometimes (score 2), often (score 3) and always (score 4). Accordingly, the sum of the scores obtained varies from 0 to 88.(6) Accordingly, the scoring of care burden of family caregivers is as follows; score between 0-20 (low or no care pressure to family caregivers), score 21-40 (moderate care pressure to family caregivers), and score 41-88 shows the severe care pressure to family caregivers.

Training procedure. At first, the subjects were divided into intervention and control groups using random method and using 4 blocks. Before the intervention, patients' demographic information questionnaire, asthma control test and quality of life questionnaire for patients in the intervention and control groups, caregivers' demographic information questionnaire, Zarit care burden questionnaire for the intervention and control groups, and lung function test (Spirometry) were completed for patients and control groups. 4 weeks after the intervention, asthma control test was completed for the patients in the intervention and control groups and lung function test (Spirometry) was performed for them. 8 weeks after the intervention, asthma control test and quality of life questionnaire for patients and control groups were completed and lung function test (Spirometry) was performed for them. The Zarit care burden questionnaire was also completed for the caregivers of the intervention and control groups.

In this study, patients and caregivers of the control group were not trained. In the intervention group, according to the pre-determined training program, the return method training was provided individually to the patient and his primary caregiver in three sessions of approximately 60 minutes at intervals of one day. The training was presented face to face, in simple and understandable language, without using special medical terms, along with PowerPoint and practical techniques. At the end of the training sessions, a training booklet related to that session was presented to patients and caregivers. Before each session, the patient and caregiver's knowledge were asked about the content of each session, and after each training session, the patient and caregiver were asked questions again to assess the individual's learning. Accordingly, the correct answer to these questions at the end of each session was the basis for completing the training. The score of the return training was determined in such a way that if the patient answered 75% of the questions correctly, it would be considered as the effectiveness of the training and otherwise the training would continue. In order to provide more guidance and support in the intervals between sessions and during the 8-week follow-up period, the researcher answered the possible ambiguities of the intervention group by phone for about 10 minutes and in accordance with the needs of the patient and his caregiver.

Content of training sessions for patients and caregivers. The content of the sessions conducted by the researcher for patients and caregivers of the intervention group included the following: (i) First session: In this session, they were taught about asthma, disease triggers and allergens, familiarity with common medications and their side effects, and ways to prevent infections and asthma attacks; (ii) Second session: in the session, the were taught about proper breathing techniques and effective coughing, motivational dialogue to quit smoking and prevent smoking, diet and complementary nutrition, an exercise in asthma, and how to properly deal with acute conditions; and (iii) Third session: In this session, the use of asthma and nebulizer, familiarity with the peak flow meter device and how to use it were taught. Also, patients were evaluated for the correct performance of the taught techniques and the contents of previous sessions.

Statistical analysis. Data were evaluated using SPSS v.19 software and the final analysis was performed on 58 patients and caregivers. Mean and standard deviation were used to describe the data. Furthermore, the frequency and percentage were used to describe the qualitative data. To comparison between the two groups, in case of parametric assumptions, the T-test of two independent samples was used and in case of no assumptions, Mann-Whitney test was used. Dependent t-pair test was used to evaluate the intra-group comparison and Wilcoxon nonparametric test was used if the hypotheses were not met. Repeated analysis test was used to compare more than two groups if parametric assumptions were made and Friedman test was used if no assumptions were made. The relationship between qualitative variables was also assessed by Chi-square test (Significance level was considered 0.05 for all tests).

## Results

Table 1 shows that the only variables studied in which statistically significant differences were found were sex and other diseases, which had higher proportions in the intervention group than in the control group.

**Figure:**
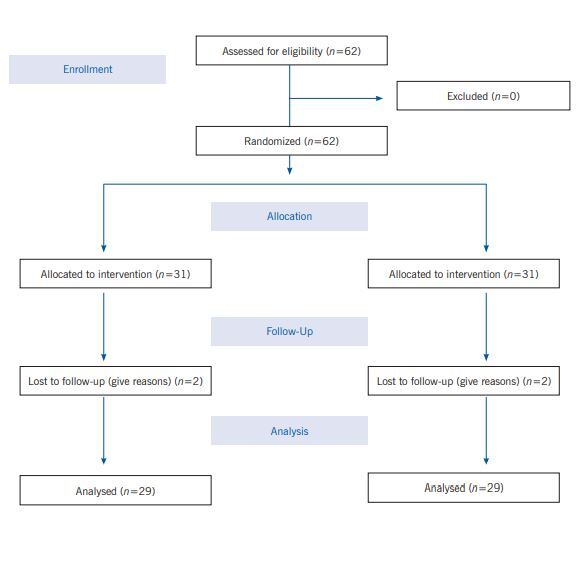

**Table. 1:** Frequency distribution of demographic variables of the studied units in the control and intervention groups

Groups	Groups	Intervention	Intervention	Control	Control	*p-value*
Variable	Variable	Number	Percentage	Number	Percentage	*p-value*
Age	18-30	1	3.4	0	0.0	0.132
Age	31-40	7	24.1	7	24.1	0.132
Age	41-50	5	17.2	11	37.9	0.132
Age	51-60	14	48.3	11	37.9	0.132
Sex	Male	3	10.3	13	44.8	0.003
Sex	Female	26	89.7	16	55.2	0.003
Level of education	Illiteratee	5	17.2	1	3.4	0.060
Level of education	Primary	6	20.7	10	34.5	0.060
Level of education	Lower than diploma	3	10.3	9	31.0	0.060
Level of education	Diploma	9	31.0	6	20.7	0.060
Level of education	Bachelor	6	20.7	2	6.9	0.060
Level of education	Upper than bachelor	0	0.0	1	3.4	0.060
Marital status	Single	3	10.3	1	3.4	0.106
Marital status	Married	20	69.0	27	93.1	0.106
Marital status	Divorced	3	10.3	0	0.0	0.106
Marital status	The wife died	3	10.3	1	3.4	0.106
Employment status	Employee	1	3.4	3	10.3	0.140
Employment status	Farmer	0	0.0	1	3.4	0.140
Employment status	Free	1	3.4	6	20.7	0.140
Employment status	housewife	20	69.0	11	37.9	0.140
Employment status	Unemployed	1	3.4	1	3.4	0.140
Employment status	Worker	1	3.4	2	6.9	0.140
Employment status	Retired	5	17.2	5	17.2	0.140
Income level	Less than 30 dollar	8	27.6	9	31.0	0.892
Income level	Between 30-60 dollar	14	48.3	12	41.4	0.892
Income level	More than 60 dollar	7	24.1	8	27.6	0.892
Having health insurance	Yes	28	96.6	28	96.6	0.754
Having health insurance	No	1	3.4	1	3.4	0.754
Habitat	City	28	96.6	27	93.1	0.079
Habitat	Village	8	27.6	2	6.9	0.079
Another diseases	Yes	18	62.1	9	31.0	0.034
Another diseases	No	11	37.9	20	69.0	0.034

### Comparison of the mean score of asthma control test in control and intervention groups

Based on the results of repeated measures test, the mean score of asthma control test was different. The trend of changes in the mean score of asthma control test in the intervention group (before intervention =14.37±4.24, 4 weeks after intervention = 17.34±4.02, 8 weeks after intervention =20.03±3.51) more than the control group (before intervention = 14.86±3.80, 4 weeks after the intervention = 13.41±3.69, 8 weeks after the intervention = 13.03±3.64 which indicates the effect of the intervention on this group (p = 0.001), the asthma control score was statistically significant between the intervention and control groups (p <0.05); but the time and time/group variables showed no significant effect on asthma control score. In other words, time had no effect on asthma control test.

### Comparison of mean scores of Spirometry indices in intervention and control groups

According to Table 2, in comparing the mean scores of Spirometry indices between the intervention and control groups before, 4 weeks after and 8 weeks after the intervention, it was found that 8 weeks after the intervention in the mandatory vital capacity index, there was a statistical difference between the control and intervention There was significance (p = 0.028). Regarding Spirometry FEV1 index, 8 weeks after the, there was a statistically significant difference between the control group and the intervention (p = 0.022). Regarding Spirometry FEV1/FVC index, 8 weeks after the intervention, there was a statistically significant difference between the control and intervention groups (p = 0.019). According the Spirometry FEF 25-75% index in 8 weeks after intervention, there was a statistically significant difference between control and intervention (p =0.023).

**Table. 2:** Comparison of mean scores of Spirometry indices by moment and groups

Spirometry	Moment	Intervention	Intervention	Control	Control	*p-value*
Spirometry	Moment	Mean	SD	Mean	SD	*p-value*
FVC	Before intervention	75.89	22.52	81.34	22.63	0.428
FVC	4 weeks after the intervention	89.79	22.12	83.55	20.32	0.268
FVC	8 weeks after the intervention	90.89	20.60	79.27	18.46	0.028
FEV1	Before intervention	66.13	22.53	74.93	23.69	0.189
FEV1	4 weeks after the intervention	80.44	20.90	74.17	22.76	0.279
FEV1	8 weeks after the intervention	85.51	21.03	70.62	22.78	0.022
FEV1/FVC	Before intervention	72.68	12.71	75.68	12.31	0.319
FEV1/FVC	4 weeks after the intervention	75.96	12.71	72.06	14.87	0.312
FEV1/FVC	8 weeks after the intervention	80.37	13.99	72	12.59	0.019
FEF 25-75%	Before intervention	48.82	31.77	61.10	35.20	0.124
FEF 25-75%	4 weeks after the intervention	57.75	29.92	58.3	32.30	0.973
FEF 25-75%	8 weeks after the intervention	67.31	28.92	49.58	28.82	0.023

### Frequency distribution of demographic variables of patients' primary caregiver

Using Chi-square and Fisher tests, it was shown that the two groups of control and intervention did not have a statistically significant difference with each other in terms of age and sex (p> 0.05). In addition, there was no statistically significant difference between the two groups of control and intervention in terms of variables of education level, marital status, patient relationship and chronic diseases (p> 0.05) (Table 3).

**Table 3:** Demographic variables of patients' primary caregiver

Groups	Groups	Intervention	Intervention	Control	Control	*p-value*
Variable	Variable	Percentage	Number	Percentage	Number	*p-value*
Age	Less than 20	10.3	3	10.3	3	0.91
Age	20-30	13.8	4	13.8	4	0.91
Age	31-40	17.3	5	20.18	6	0.91
Age	41-50	13.8	4	20.18	6	0.91
Age	51-60	27.6	8	27.6	8	0.91
Age	61-70	13.8	4	3.4	1	0.91
Age	More than 70	3.4	1	3.4	1	0.91
Sex	Male	48.3	14	48.3	14	0.793
Sex	Female	51.7	15	51.7	15	0.793
Level of education	illiterate	10.3	3	10.3	3	0.955
Level of education	Lower than diploma	44.8	13	48.3	14	0.955
Level of education	Diploma	27.6	8	31.1	9	0.955
Level of education	College education	17.3	5	10.3	3	0.955
Marital status	Single	17.2	5	13.8	4	0.219
Marital status	Married	69	20	86.2	25	0.219
Marital status	Divorced	6.9	2	0	0	0.219
Marital status	The wife died	6.9	2	0	0	0.219
Relationship with the patient	Father	10.3	3	3.4	1	0.260
Relationship with the patient	Mother	0	0	3.4	1	0.260
Relationship with the patient	Wife	51.7	15	72.5	21	0.260
Relationship with the patient	Sister	3.4	1	3.4	1	0.260
Relationship with the patient	Brother	0	0	0	0	0.260
Relationship with the patient	Child	34.6	10	17.3	5	0.260
Chronic diseases	Yes	24.1	7	31	9	0.770
Chronic diseases	No	75.9	22	69	20	0.770

### Comparison of mean scores of care stress in control and intervention groups

Mann-Whitney test was used to compare pre-intervention care pressure in the two groups due to non-normality of data. According to the results, there was no statistically significant difference between the two groups in terms of mean score of care pressure (mean care pressure of the control group= 35.24±10.73 and the intervention group = 35.51±11.31; p = 0.75). To compare the care pressure 8 weeks after the intervention in the two groups, due to the normal distribution of data, t-test was used and the results showed that there was a statistically significant difference between the two groups in terms of mean care pressure score (The mean care pressure of the control group = 35.72±9.63 and 18 ±6.61 in the intervention group (p<0.001). In other words, the Tech-back training method has reduced the care pressure in the caregivers of the intervention group.

## Discussion

Today, no one doubts the need for disease prevention to take precedence over treatment, because any disease, in addition to the suffering it imposes, requires a high cost to cure it, which affects both the individual and the community, as a result, it can be helpful to teach disease-related content in a way that stays in the mind for a long time and promotes better interaction between the patient and the nurse. Although some diseases, such as asthma, are not curable, they can be controlled. In this study, we used the Teach-back education method for patients with asthma and measured its effect on patients' asthma control and family care pressure in patients with asthma.

Based on the results, in comparing the mean scores of asthma control test in the control and intervention groups, it was found that the mean scores of asthma control test in the control group, before, 4 weeks after and 8 weeks after the intervention were 14.86, 13.41, and 13.03 respectively. Accordingly, the results of the control group showed that their disease was not under control in all 3 time periods. On the contrary, our training method on the intervention group led to disease control in patients in this group. After 8 weeks, the patients in the intervention group went from uncontrolled asthma to controlled asthma. Due to the significance of the group variable, there was a statistically significant difference between the intervention and control in terms of asthma control. In addition, the time and time / group variables showed no significant effect on asthma control score. These findings indicate the effectiveness of Teach-back education in controlling asthma.

Furthermore, in comparing the mean scores of Spirometry indices, it was found that the mean scores of all Spirometry indices in the intervention group were statistically significant and not the same. Also, the trend of changes in the mean of these scores showed that this educational method has a positive effect on patients and their Spirometry index has increased.

Shermans et al.(14) conducted a randomized controlled clinical trial with the aim of the effect of a 10-minute training session on patients' asthma control. In the intervention group, a 10-minute training including basic information about asthma and the effectiveness of medications was given. This training was not provided to the control group. The training session caused that after 3 months, asthma was significantly controlled in the intervention group compared to the control group. However, in the study of Arikan-Yildiz et al.(15) the scores of asthma control test were not significantly different in comparison before and after the intervention; which did not agree with the results of the study. The reasons for this difference in results include differences in the number of training program sessions (one hour training session), the age range of study participants (5-18 years), the manner of holding training sessions or the educational content presented in the sessions

In comparing the mean scores of caregiving stress in the two groups of control and intervention before and after the intervention, it was found that there was no statistically significant difference between the two groups in terms of mean scores of caregiving pressure before the intervention; But 8 weeks after the intervention, there was a statistically significant difference between the two groups in terms of mean care pressure score. Ghaneh et al.(16) conducted a study to determine the effect of supportive education program on the care pressure of family caregivers of patients undergoing hemodialysis. The results showed that the care pressure in the experimental group decreased after the intervention. According to the results of this study, it can be said that the use of educational-supportive programs can be effective in reducing the care pressure of family caregivers.

In conclusion, the results of the present study showed that teaching asthma related topics to the patient and her caregiver along with the follow-up and supervision of the nurse improves asthma control and also reduces the caregiver pressure. Additionally, this study also shows that the use of educational methods by asthmatics in a way that stays in the mind for a long time leads to an improvement in their condition. Nursing managers should inform staff about the importance and learning of these training methods by holding training courses. It is also suggested that more educational-therapeutic methods be used to cure diseases.
